# Mid-vastus total knee arthroplasty for medial osteoarthritis recovers gait balance control better than lateral parapatellar approach three months post-surgery

**DOI:** 10.3389/fbioe.2023.1133992

**Published:** 2023-03-22

**Authors:** Pei-An Lee, Ting-Ming Wang, Yu-Ting Chen, Kuan-Hsien Wu, Hwa-Chang Liu, Tung-Wu Lu

**Affiliations:** ^1^ Department of Biomedical Engineering, National Taiwan University, Taipei, Taiwan; ^2^ Department of Orthopaedic Surgery, School of Medicine, National Taiwan University, Taipei, Taiwan; ^3^ Department of Orthopaedic Surgery, National Taiwan University Hospital, Taipei, Taiwan; ^4^ Department of Orthopaedic Surgery, Taiwan Adventist Hospital National Taiwan University Hospital, Taipei, Taiwan

**Keywords:** mid-vastus approach, lateral parapatellar approach, total knee arthroplasty, balance, motion analysis

## Abstract

Total knee arthroplasty (TKA) approaches affect recovery outcomes, with different levels of residual loss of muscle strength and functional deficits. The current study compared the gait balance control in older individuals 3 months after TKA *via* the lateral parapatellar approach (LPPA) and mid-vastus approach (MVA) in terms of the inclination angle (IA) of the center of pressure (COP) to the body’s center of mass (COM) vector, and the rate of change of IA (RCIA). In a gait laboratory, 12 patients with severe medial knee osteoarthritis who had undergone bilateral TKA *via* LPPA and 12 *via* MVA were evaluated and compared against 12 healthy controls for their balance control during gait 3 months after surgery. The participants’ kinematic data and ground reaction forces were measured synchronously using an 8-camera motion capture system and three forceplates, respectively, from which the COM, COP, IA and RCIA were calculated using a 13-body-segment model. The LPPA group showed significantly greater sagittal IA during DLS (*p* < 0.01) but less sagittal and frontal RCIA throughout the gait cycle (*p* < 0.04) compared to controls. The MVA showed better recovery in the balance control with most IA and RCIA variables similar to those of the healthy controls throughout the gait cycle. The patients with LPPA walked with a compromised balance control throughout the gait cycle while the MVA group showed close-to-normal balance control with a slight decrease in sagittal RCIA during SLS. The current between-approach findings were likely related to the differences in the muscles involved during surgery, suggesting that MVA may be a better choice than LPPA when taking short-term gait balance control into consideration.

## Introduction

Knee osteoarthritis (OA) is the most common joint disorder, affecting more than 654.1 million adults worldwide in 2020 (Cui et al., 2020). Over 10% of adults aged above 60 years suffer from knee OA (Zhang and Jordan, 2010), with limited range of motion, pain and joint stiffness, reduced physical function (Yelin et al., 1987; Jinks et al., 2007; Cubukcu et al., 2012), and affected gait patterns and balance control, resulting in an elevated risk of falling (Levinger et al., 2012; Tsonga et al., 2015). Total knee arthroplasty (TKA) has been the main treatment for advanced knee OA, showing notable efficacy in reducing pain and improving the motion and loadings of the operated knee during walking (Hatfield et al., 2011; Orishimo et al., 2012). With the advance in total knee prosthesis design for wear resistance and low failure rate, improvement of functional performance and reduction of fall risks have become a major consideration in TKA (Moutzouri et al., 2017). Different TKA surgical procedures were found to have different recovery outcomes, with different levels of loss of muscle strength and functional deficits in the lower extremities (Migliorini et al., 2020).

The midline medial parapatellar approach (MPPA) has successfully been used for TKA in the last decades (Tarazi et al., 2019), but damage to the quadriceps tendon and the extensor mechanism has been found to affect the functional outcomes (Boerger et al., 2005). More recent modifications to this traditional approach (MPPA), such as the lateral parapatellar approach (LPPA) (Keblish, 1991) and the mid-vastus approach (MVA) (Engh et al., 1997), are believed to give better functional outcomes (Masjudin and Kamari, 2012; Gunst et al., 2016). The LPPA involves an incision on the mid-line of the quadriceps tendon (about 5 cm proximal to the apex on the patella) to separate the vastus lateralis from the remainder of the quadriceps down to the base of the patella, around the lateral border of the patella, and finally along the lateral side of the patellar tendon (Liu et al., 2001). The LPPA allows minimal muscle damage, preserves medial blood supply, and improves patellar tracking (Cristea et al., 2016). The MVA was also developed to reduce disruption of the extensor mechanism keeping the quadriceps tendon intact. The vastus medialis oblique muscle belly is split in the direction of its fibers, from a point at the superior-medial border of the patella and extending medially towards the inter-muscular septum (Dalury and Jiranek, 1999; Liu H.-W. et al., 2014). The incision then continues from the superior-medial border of the patella, around the medial border of the patella, and finally along the medial side of the patellar tendon (Engh et al., 1997). This approach has been shown to provide short recovery time and better radiological results than the traditional approach (Mehta et al., 2017). Both LPPA and MVA are reported to be superior to the traditional MPPA technique with higher tissue preservation and faster recovery (Sekiya et al., 2014; Mehta et al., 2017) but the functional outcomes between LPPA and MVA surgical approaches have not been well documented. Since LPPA and MVA affect different muscles and soft tissues, identifying their effects on the functional performance will help provide guidelines for surgical approach selections for individual patients.

It has been reported that older people with knee OA are at higher risk of loss of balance during level walking and remain so after TKA (Kearns et al., 2008; Swinkels et al., 2009; Matsumoto et al., 2012; Chan et al., 2018; Lee P. A. et al., 2021). While full recovery of whole-body balance control can be expected 1 year after TKA (Lee P. A. et al., 2021), it is important to avoid falls, especially during the first 3 months of recovery following TKA (Swinkels et al., 2009; Matsumoto et al., 2012; Lee P. A. et al., 2021). Since the muscular system plays an important role in whole-body balance during activities (Fukagawa et al., 1995; Daubney and Culham, 1999), any reduction in the muscle strength at the knee will have a direct effect on the normal contribution of the knee in supporting the lower extremities’ maintenance of whole-body balance. For example, patients with bilateral medial knee OA were found to have reduced knee extensor moments and contributions to the support moments needed to prevent collapse of the lower limbs while balancing and supporting the body (Liu Y.-H. et al., 2014). Similarly, it is expected that the amount of damage to the surrounding muscles during surgery will have a direct effect on the mechanics of the knee and the normal function of the lower extremities in maintaining balance. The LPPA and MVA may have different effects on gait balance during the early recovery phase as the LPPA involves incisions to the quadriceps tendon and vastus lateralis muscles, while the MVA reduces the damage to the quadriceps tendon and vastus medialis by splitting the vastus medialis obliquus. Such potential differences may be a factor that should be considered in surgical decision-making. Previous studies have compared the clinical results of MVA or LPPA separately with the MPPA (Dalury and Jiranek, 1999; Liu H.-W. et al., 2014; Heekin and Fokin, 2014; Nutton et al., 2014) but none have reported direct comparisons of the clinical outcome between MVA and LPPA. Studies on whole-body balance control during gait in patients before and after TKA have been limited and have not specified the surgical approaches used (Mandeville et al., 2008; Lee P. A. et al., 2021). Thus, the effects of the TKA surgical approach on balance control during gait remains unexplored.

The balance control during gait can be quantified and evaluated by the motions of the body’s center of mass (COM) relative to the center of pressure (COP) in terms of the inclination angle (IA) and the rate of change of IA (RCIA) of the COM-COP vector (Huang et al., 2008; Chien et al., 2013; Lee P.-A. et al., 2021). These variables—especially the frontal plane components—have been shown to be effective in distinguishing unbalanced patients from healthy controls during level walking (Chou et al., 2003; Lee and Chou, 2006) and have high test-retest reliability (De Jong et al., 2020). The IA and RCIA analysis has been used in studies on various populations during various dynamic activities (Huang et al., 2008; Chien et al., 2013; Lee P.-A. et al., 2021). Bilateral severe medial compartment knee OA has been shown to compromise the COM-COP control in older adults during gait, which may be related to the increased risk of falling in this population (Lee P.-A. et al., 2021). Comparisons of the IA and RCIA variables between LPPA and the MVA with respect to healthy controls enable a quantitative analysis of the effects of surgical approaches, i.e., LPPA and MVA, on the dynamic balance control during gait following TKA. Such information will provide useful insights for the selection of a surgical approach and the management of fall risks in patients with TKA, especially during the early phase of recovery.

The purpose of the current study was to identify and compare the whole-body balance control during level walking in older people 3 months after TKA *via* LPPA vs. MVA, in terms of IA and RCIA. It was hypothesized that both patient groups would show compromised balance control with greater IA but less RCIA when compared to healthy controls, and that patients who underwent TKA *via* MVA would show better whole-body balance control than patients with LPPA as compared to healthy controls.

## Materials and methods

### Participants

**FIGURE 1:**
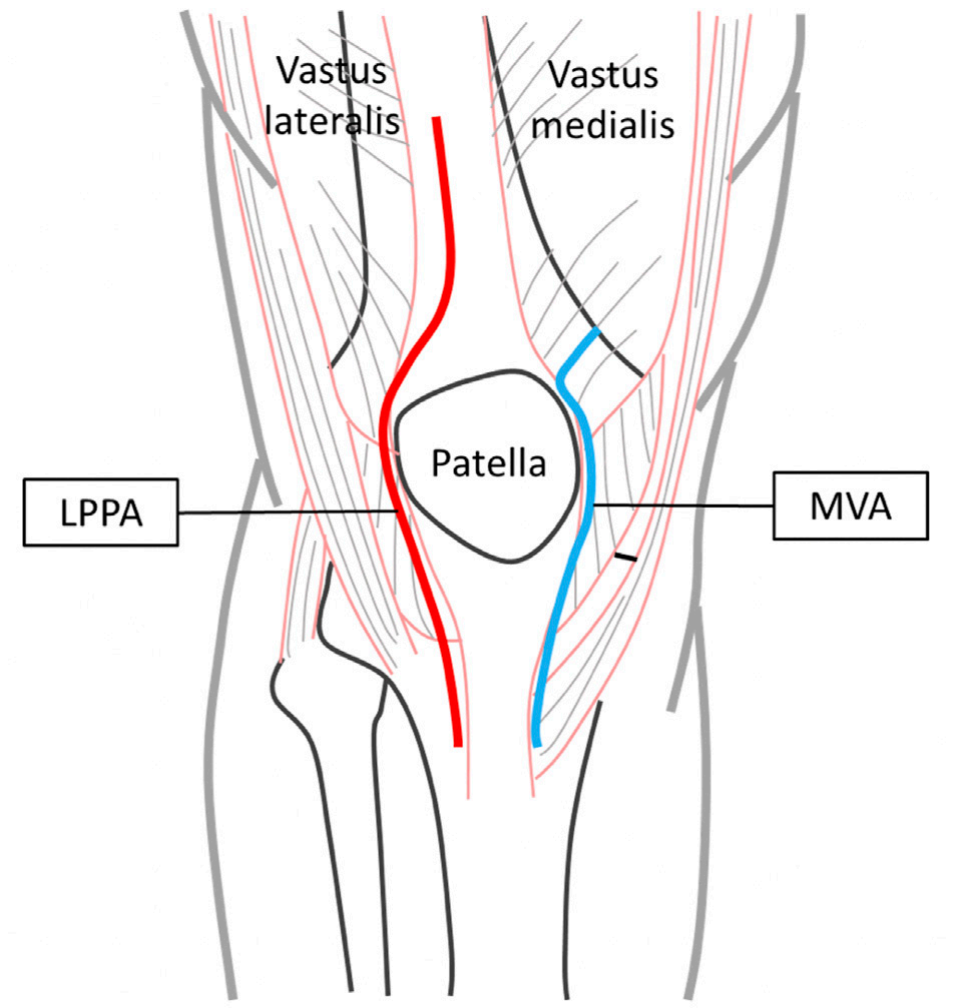
Surgical approaches for TKA—lateral parapatellar approach (LPPA, red line) and mid-vastus approach (MVA, blue line).

All experiments and procedures of the current study were conducted with the approval of Taiwan Adventist Hospital Institutional Review Board (IRB No. 106-E-15), conforming to the Ethical Principles for Medical Research Involving Human Subjects (World Medical Association Declaration of Helsinki). Twelve older adults with severe bilateral medial knee OA who had undergone TKA *via* LPPA (LPPA group; male/female: 2/10; age: 67.3 ± 6.8 years, height: 157.7 ± 6.8 cm, mass: 71.0 ± 14.9 kg, BMI: 28.4 ± 4.1, tibiofemoral angle: −2.2 ± 7.9°) and 12 *via* MVA (MVA group; male/female: 2/10; age: 68.6 ± 7.8 years, height: 155.5 ± 6.0 cm, mass: 64.1 ± 13.3 kg, BMI: 26.5 ± 5.5, tibiofemoral angle: 2.5 ± 4.5°) for treating severe bilateral anteromedial knee OA (KL grade 4) participated in the current gait study with written informed consent. The tibiofemoral angles were measured as the angle between the anatomic femoral axis and the mechanical limb axis on an anterioposterior radiograph of the lower limbs (Ishii et al., 1995). Twelve healthy controls (Control group; male/female: 2/10; age: 67.4 ± 6.1 years, height: 156.7 ± 6.0 cm, mass: 65.5 ± 8.8 kg, BMI: 26.7 ± 3.7) were selected from the local community to match with the patient groups for sex, age and BMI. The participants with TKA met the following including criteria: 1) radiographically confirmed severe bilateral medial knee OA with affected varus alignment (Kellgren and Lawrence grade 4); 2) 3 months after bilateral TKA *via* LPPA or MVA; and 3) independent walking without assistive device. All participants were free from neuromusculoskeletal diseases or pathology that might affect gait. The patients were implanted bilaterally with cemented posterior stabilized total knee prostheses (NexGen^®^Legacy^®^ Posterior Stabilized-Flex, Zimmer Biomet, United States) *via* the mid-vastus approach (MVA) (Dalury and Jiranek, 1999) or *via* the lateral aspect of the patella (LPPA) (Figure 1). All surgical procedures were performed by a senior consultant orthopedic surgeon (HCL) specializing in knee surgery with more than 30 years of experience. The LPPA and MVA groups received equal in-patient rehabilitation after surgery for at least 1 week, focusing mainly on joint mobilization and muscle strengthening, including passive/active flexion/extension, isometric quadriceps contractions, inner range quadriceps exercises and straight leg raises. They were taught a standardized home-exercise program before they left the hospital, including simple exercises to retrain lower-limb strength and to increase knee mobility. All participants in the LPPA and MVA groups were assessed radiographically and *via* the Western Ontario and McMaster Universities Osteoarthritis Index (WOMAC) questionnaire 3 months after TKA. An *a priori* power analysis using G*POWER (Erdfelder et al., 1996) based on pilot results on IA and RCIA from five participants per group determined that a projected sample size of seven participants for each group would be needed for comparisons among LPPA, MVA and Control groups using one-way ANOVA with a power of 0.8 and a Cohen’s *f* = 0.75 at a significance level of 0.05. Thus, 12 participants for each group were appropriate for the study’s main objectives.

In a hospital gait laboratory, each participant walked at her/his preferred walking speed on a 10-m walkway while the ground reaction forces (GRF) were measured at 1,080 Hz using three forceplates (50.8 × 46.2 cm, OR-6-7-1,000, AMTI, United States) and the three-dimensional (3D) motions of the body segments were measured at 120 Hz using an 8-camera motion analysis system (Vicon MX T-40, United Kingdom) with 54 infrared retro-reflective markers placed on specific anatomical landmarks commonly used in human motion analysis (Chen et al., 2011). The participants were allowed to walk on the walkway several times before data collection. Data from a total of six successful trials with each foot placed on one forceplate, each containing a complete gait cycle for each lower limb, were obtained for each participant.

**FIGURE 2:**
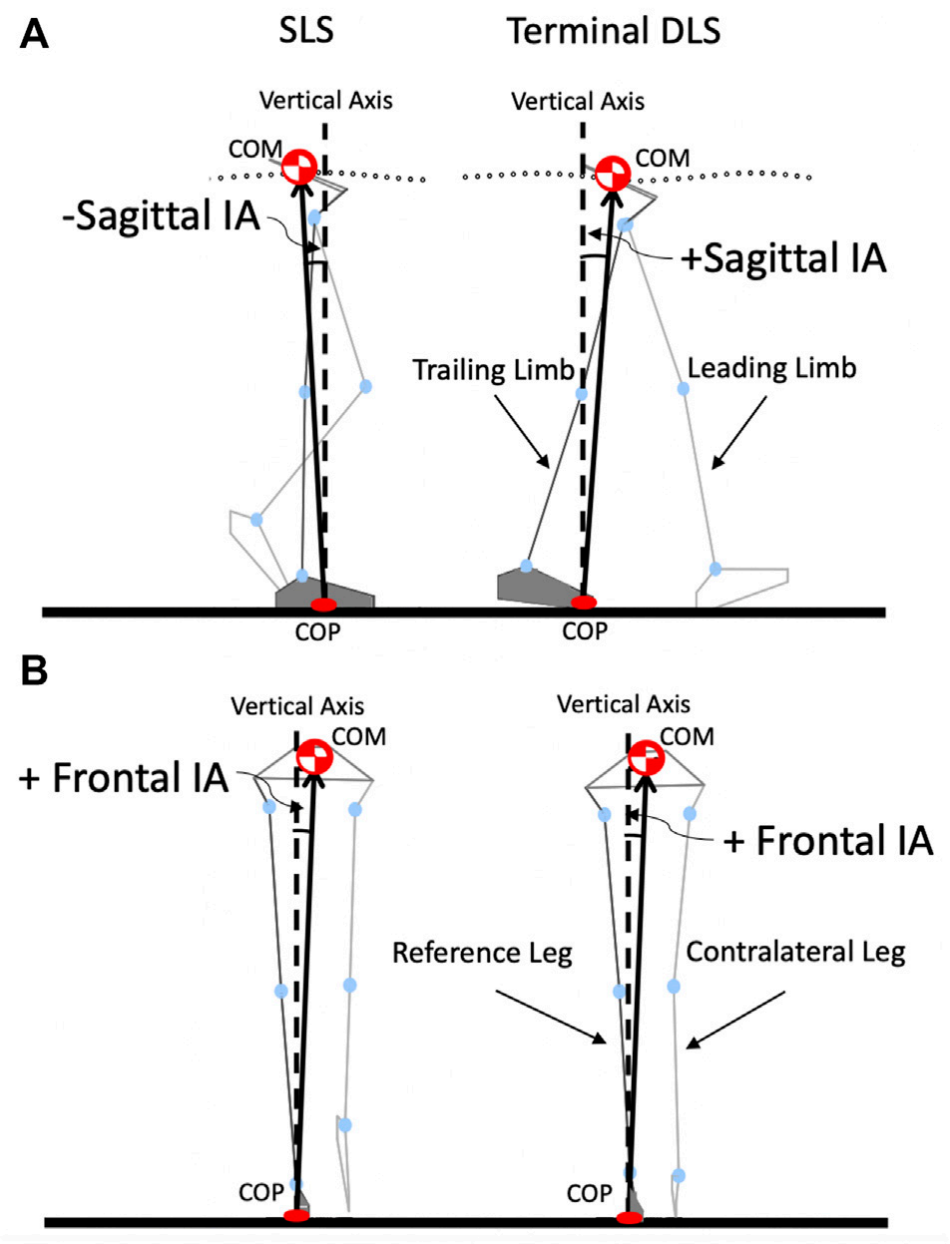
Schematic diagram showing a stick figure of a typical participant during level walking showing the COM and COP, and the COM-COP vector forming the sagittal inclination angle (IA) **(A)** and frontal IA **(B)** with the vertical. The reference limb is shown in darker grey.

With the measured GRF and marker data, the mass and position of the COM for each body segment were obtained using an optimization-based method, which has been shown to have better accuracy than traditional prediction methods (Chen et al., 2011). The whole body’s COM during gait was then calculated as the mass-weighted sum of all the segmental COM position vectors using a 13-body-segment model (Lee and Chou, 2006; Mandeville et al., 2008; Lu et al., 2017; Lee p.-A. et al., 2021; Lee et al., 2022), and the corresponding COP position was calculated using the forceplate data. The inclination angles (IA) of the COM-COP vector in the sagittal and frontal planes were then calculated according to Hsu et al. (2010):

$$\vec{t}=\left(\vec{Z}\times \frac{{\vec{P}}_{COM-COP}}{\left|{\vec{P}}_{COM-COP}\right|}\right)$$ (1)

$$Sagittal\;IA=\sin^{-1}\left(t_{Y}\right)$$ (2)

$$Frontal\;IA=\left\{\begin{array}{c} {-\sin}^{-1}\left(t_{X}\right),for\;the\;right\;limb\\ \sin^{-1}\left(t_{X}\right),for\;the\;left\;limb \end{array}\right.$$ (3)

where ${\vec{P}}_{COM-COP}$ was the vector pointing from the COP to the COM, $\vec{Z}$ was the vertical unit vector, and $\vec{X}$ was the unit vector pointing to the direction of progression. The corresponding RCIA were calculated by smoothing and differentiating the IA trajectories using the GCVSPL package (Woltring, 1986). A positive sagittal IA indicates a COM position anterior to the COP, while a positive frontal IA indicates a COM position away from the COP and towards the contralateral limb (Figure 2). At a standing posture, the greater the magnitude of the IA, the greater the COM is away from the COP and the greater muscular efforts (joint moments) needed to maintain the posture. During walking, even if the COM is outside the base of support, with an appropriate IA-RCIA combination, a person can still tolerate and regain balance without initiating a fall, according to the position and velocity control of the COM described by Pai and Patton (Pai and Patton, 1997). In other words, the greater the IA, the more the COM-COP vector deviates from vertical and the greater the muscular effort needed to bring the COM back to be above the COP, unless the RCIA is towards the vertical (i.e., negative RCIA for positive IA; positive RCIA for negative IA). Thus, the data of IA and RCIA should be considered together to identify the deviations and/or strategies of dynamic balance control as the position and velocity of the COM together determine the safe region without initiating a fall (Pai and Patton, 1997). A person with greater than normal IA but without an appropriate RCIA is considered to have compromised balance control, while a less than normal IA and RCIA indicate a conservative balance strategy. The current data analyses and graphics generation were conducted using in-house developed programs in MATLAB (R2017b, MathWorks, United States) (Wu et al., 2020; Lee P. A. et al., 2021; Lee et al., 2022).

For statistical analysis, the values of the sagittal and frontal IA and RCIA at heel-strike and toe-off were obtained for both limbs, for each trial and each participant. The time-averaged values and ranges of IA and RCIA over the phases of double-limb support (DLS) and swing/single-limb support (SLS) were also obtained. For each variable, data from the six trials were averaged. Each variable was first tested for normality using a Shapiro-Wilk test. For variables of normal distribution, a one-way analysis of variance (ANOVA) was performed between MVA, LPPA, and Control groups. Once a significant main effect was found, *post hoc* tests were performed using an independent *t*-test. All significance levels were set at α = 0.05. All statistical analyses were performed using SPSS version 20 (SPSS Inc., Chicago, IL, United States).

## Results

No significant differences in age, height, mass or BMI were found among the LPPA, the MVA and Control groups. Compared to the Control, the LPPA and MVA groups showed greater WOMAC scores in pain, stiffness and physical function with smaller walking speed and cadence but greater step width (Table 1). No significant differences between the LPPA and the MVA groups were found in WOMAC scores or temporo-spatial parameters.

**TABLE 1:** Means (standard deviations) of the WOMAC scores and spatiotemporal parameters during walking in the LPPA, MVA, and Control groups. *p*-values for comparisons between patient and Control groups using independent *t*-tests are also given.

	LPPA	MVA	Control	P_G_ (P_LC_, P_MC_, P_LM_)
WOMAC scores				
Pain	12.9 (11.4)	17.5 (16.9)	4.2 (3.6)	**0.02**, **0.02**, 0.44
Stiffness	35.4 (14.9)	38.5 (24.1)	6.3 (6.5)	**<0.01**, **<0.01**, 0.71
Physical function	19.0 (11.9)	22.9 (19.3)	4.3 (6.6)	**<0.01**, **0.01**, 0.56
Spatiotemporal parameters				
Walking speed (mm/s)	727.7 (153.1)	756.4 (122.0)	871.7 (60.4)	**0.01**, **0.01**, 0.62
Cadence (steps/min)	90.2 (15.2)	93.1 (8.5)	105.0 (7.8)	**0.01**, **<0.01**, 0.57
Stride length (mm)	967.3 (55.8)	972.9 (74.4)	1,001.2 (49.8)	Main Effect: 0.87
Step length (mm)	479.0 (31.3)	481.3 (38.0)	494.6 (29.2)	Main Effect: 0.74
Step width (mm)	127.3 (27.6)	131.0 (15.6)	103.3 (26.4)	**0.04**, **0.01**, 0.69

Bold values are significantly different from the groups.

P_G_, group effect; P_LC_, LPPA, vs. Control; P_MC_, MVA, vs. Control; P_LM_, LPPA, vs. MVA.

**FIGURE 3:**
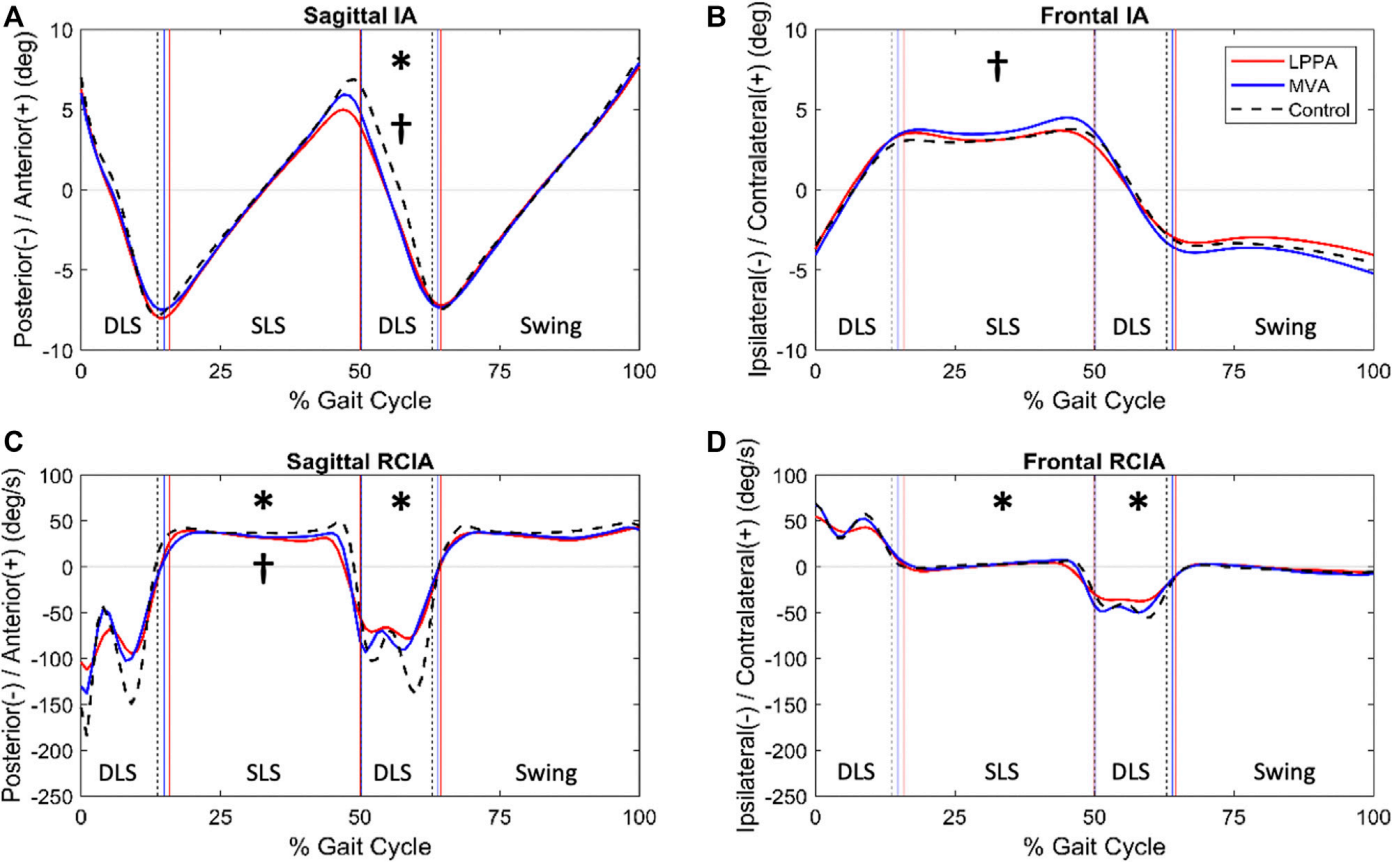
Mean curves of the COM-COP inclination angles (IA) and their rates of change (RCIA) in the sagittal and frontal planes for the knee osteoarthritis groups 3 months after TKA *via* LPPA (red lines) and MVA (blue lines), and the control group (black dashed lines) during level walking in the sagittal **(A,B)** and frontal **(C,D)** planes. Positive sagittal and frontal IA indicate COM positions that are anterior and contralateral to the COP, respectively. Positive sagittal and frontal RCIA indicate rates of anterior changes and contralateral changes in the corresponding IA, respectively. Heel-strike (HS), contralateral toe-off (CTO), contralateral heel-strike (CHS) and toe-off (TO) are indicated by vertical lines. IA and RCIA were temporally normalized by the stride time to be in 0%–100% of the gait cycle. Asterisks (*) indicate statistical significance (*p* < 0.05) between LPPA and Control, and crosses (†) indicate statistical significance (*p* < 0.05) between MVA and Control for the time-averaged IA or RCIA values over double-limb support (DLS) or single limb-support (SLS) phases.

**FIGURE 4:**
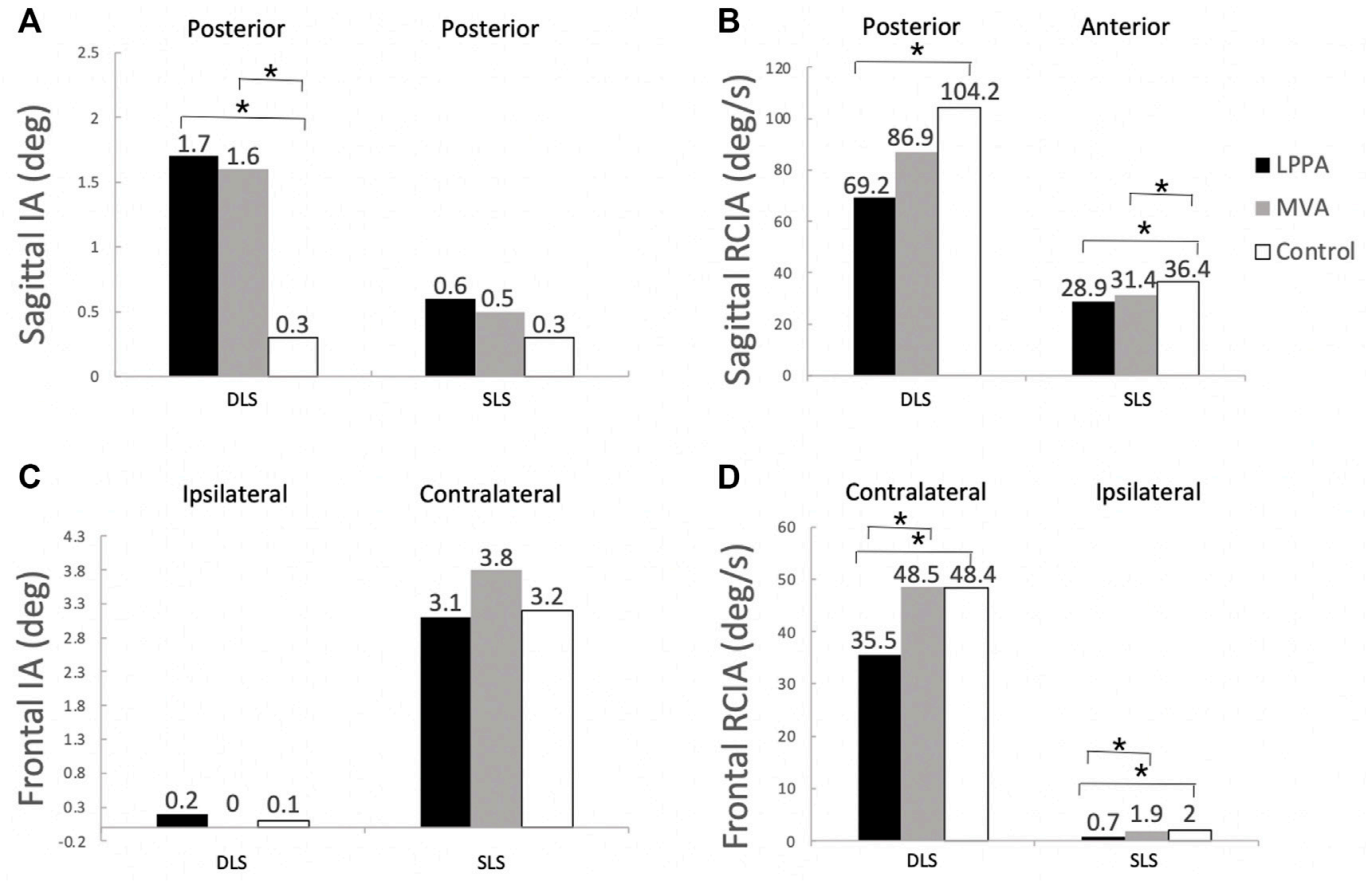
Mean values of time-averaged COM-COP inclination angles (IA) and their rates of change (RCIA) over phases of terminal double-limb support (DLS) and single-limb support (SLS) during level walking in the sagittal **(A,B)** and frontal **(C,D)** planes for the lateral parapatellar approach (LPPA), mid-vastus approach (MVA), and the control groups. Positive sagittal and frontal IA indicate COM positions that are anterior and contralateral to the COP, respectively. Positive sagittal and frontal RCIA indicate rates of anterior changes and contralateral changes in the corresponding IA, respectively. Asterisks indicate statistical significance (*p* < 0.05).

Compared to Control, the LPPA group showed significantly greater average IA during DLS but with less range of IA and average RCIA throughout the gait cycle in the sagittal plane (Table 2) (Figures 3, 4). In the frontal plane, the LPPA also showed significantly less average RCIA throughout the gait cycle (Table 3) (Figures 3, 4). On the other hand, the MVA group showed significantly greater average IA during DLS but significantly less average RCIA during SLS in the sagittal plane (Table 2) (Figures 3, 4). In the frontal plane, the MVA group showed significantly greater average IA during SLS (Table 3) (Figures 3, 4).

**TABLE 2:** Means (standard deviations) of inclination angles (IA) and rates of change of IA (RCIA) in the sagittal plane at heel-strike (HS) and toe-off (TO), and their average values and ranges during double-limb support (DLS) and single-limb support (SLS), as well as peak RCIA during DLS. *p*-values are also given for comparisons between groups using one-way analysis of variance (ANOVA) and for *post hoc* analysis using independent *t*-tests.

	LPPA	MVA	Control	P_G_ (P_LC_, P_MC_, P_LM_)
**Sagittal IA (deg)**				
Events				
HS	6.6 (0.9)	6.3 (1.4)	7.3 (0.9)	**0.03**, 0.06, 0.58
TO	−8.0 (0.7)	−8.3 (0.8)	−8.0 (0.8)	Main Effect: 0.24
Averages				
SLS	−0.6 (0.5)	−0.5 (0.6)	−0.3 (0.8)	Main Effect: 0.61
DLS	−1.7 (1.0)	−1.6 (0.5)	−0.3 (0.6)	**<0.01**, **<0.01**, 0.62
Ranges				
SLS	13.5 (2.0)	15.4 (1.7)	16.3 (1.1)	**<0.01**, 0.13, **0.02**
DLS	13.5 (1.5)	15.1 (1.3)	15.5 (1.4)	**<0.01**, 0.49, **0.01**
**Sagittal RCIA (deg/s)**				
Events				
HS	−64.0 (71.8)	−106.7 (93.6)	−131.3 (63.4)	**0.02**, 0.46, 0.22
TO	−6.5 (27.1)	−8.7 (22.7)	−36.8 (31.2)	**<0.01**, **0.02**, 0.15
Averages				
SLS	28.9 (6.0)	31.4 (4.6)	36.4 (3.3)	**<0.01**, **0.01**, 0.26
DLS	−69.2 (21.5)	−86.9 (23.2)	−104.2 (20.8)	**<0.01**, 0.07, 0.07
Peak Values				
DLS	−139.8 (50.0)	−149.7 (33.1)	−155.8 (32.4)	Main Effect: 0.61
Ranges				
SLS	115.3 (55.2)	178.0 (80.3)	175.5 (84.4)	0.05, 0.94, **0.04**
DLS	221.4 (72.2)	253.0 (68.4)	245.6 (37.6)	Main Effect: 0.43

Bold values are significantly different from the groups.

*p*-Values: P_G_ , group effect; P_LC_ = LPPA, vs. Control; P_MC_ = MVA, vs. Control; P_LM_ = LPPA, vs. MVA.

**TABLE 3:** Means (standard deviations) of inclination angles (IA) and rates of change of IA (RCIA) in the frontal plane at heel-strike (HS) and toe-off (TO), and their average values and ranges during double-limb support (DLS) and single-limb support (SLS), as well as peak RCIA during DLS. *p*-values are also given for comparisons between groups using one-way analysis of variance (ANOVA) and for *post hoc* analysis using independent *t*-tests.

	LPPA	MVA	Control	P_G_, (P_LC_, P_MC_, P_LM_)
**Frontal IA (deg)**				
Events				
HS	−3.9 (0.8)	−4.3 (1.0)	−3.8 (0.9)	Main Effect: 0.29
TO	−3.4 (0.7)	−4.0 (0.7)	−3.3 (0.9)	Main Effect: 0.13
Averages				
SLS	3.1 (0.5)	3.8 (0.6)	3.2 (0.7)	0.55, **0.04**, **<0.01**
DLS	−0.2 (0.5)	−0.0 (0.3)	0.1 (0.6)	Main Effect: 0.42
Ranges				
SLS	1.1 (0.4)	1.6 (0.5)	1.3 (0.6)	0.53, 0.26, **0.04**
DLS	6.9 (1.2)	8.6 (1.6)	7.1 (1.6)	0.80, **0.03**, **0.01**
**Frontal RCIA (deg/s)**				
Events				
HS	38.5 (31.8)	55.8 (44.0)	54.6 (21.2)	Main Effect: 0.38
TO	−8.9 (11.7)	−15.5 (9.9)	−26.0 (14.8)	**<0.01**, 0.05, 0.15
Averages				
SLS	0.7 (0.9)	1.9 (1.3)	2.0 (1.6)	**0.03**, 0.94, **0.01**
DLS	−35.5 (10.9)	−48.5 (12.3)	−48.4 (15.3)	**0.03**, 0.98, **0.01**
Peak Values				
DLS	−62.3 (23.0)	−71.6 (17.5)	−61.7 (19.3)	Main Effect: 0.41
Ranges				
SLS	49.2 (26.5)	80.4 (31.4)	70.0 (42.6)	0.17, 0.50, **0.02**
DLS	89.7 (37.9)	108.1 (29.4)	85.5 (25.3)	Main Effect: 0.19

Bold values are significantly different from the groups.

*p*-Values: P_G_ , group effect; P_LC_ = LPPA, vs. Control; P_MC_ = MVA, vs. Control; P_LM_ = LPPA, vs. MVA.

Compared to LPPA, the MVA showed similar averaged IA but with significantly greater ranges throughout the gait cycle and significantly greater ranges of RCIA during SLS in the sagittal plane (Table 2) (Figures 3, 4). In the frontal plane, the MVA group showed significantly greater ranges and average values of IA and RCIA during SLS and significantly greater ranges of IA and average RCIA during the rest of the gait cycle (Table 3) (Figures 3, 4).

## Discussion

The current study aimed to identify and compare the whole-body balance control during level walking in older people 3 months after TKA *via* LPPA or MVA, in terms of COM-COP IA and RCIA. The patients with LPPA showed a compromised balance control both during weight transfer and single-limb support as indicated respectively by the significantly greater average IA with less average RCIA during DLS, and significantly less average RCIA in both sagittal and frontal planes during SLS (Tables 2, 3). In contrast, patients with MVA showed better recovery in the COM-COP control with most IA and RCIA variables similar to those of the healthy controls throughout the gait cycle, except for greater sagittal IA during DLS, and less sagittal RCIA and greater frontal IA during SLS. Since no differences were found between LPPA and MVA in the residual pain, stiffness and physical function as indicated by the WOMAC scores, the observed differences in balance control were more likely to be related to the differences between the surgical approaches, especially the muscles involved. The current results suggest that MVA may be a better choice than LPPA for TKA when taking gait balance control into consideration.

The patients with LPPA walked at a reduced walking speed with a compromised COM-COP control both during weight transfer and single-limb support, mainly associated with significantly less RCIA in both the sagittal and frontal planes when compared with healthy controls. During DLS, the LPPA group exhibited significantly greater IA in the sagittal plane but with less RCIA in both the sagittal and frontal planes. Without an appropriate RCIA to match the greater or normal IA, the observed deviations in the COM-COP control were considered unfavourable to the dynamic balance control in this group (Pai and Patton, 1997). During DLS of normal level walking, the main task of the locomotor system is to transfer the body weight medially and anteriorly from the trailing limb to the leading limb. The COM-COP motion in this phase can be regarded as a pendulum, with the COP moving from the trailing towards the leading limb while the COM is controlled within a relatively small range. The greater IA magnitude—but without correspondingly greater RCIA–may indicate a reduced forward momentum and ability to maintain the dynamic stability of the COM in the sagittal plane with a greater risk of loss of balance (Pai and Patton, 1997; Lee P.-A. et al., 2021; Lee et al., 2022). These findings are clinically relevant as about 51% of falls by the elderly result from inadequate body weight transfer (Robinovitch et al., 2013). A significantly greater step width further indicated a compromised balance control during the preceding SLS and DLS, further affecting the subsequent SLS. During SLS, in contrast to DLS, the COM-COP motion can be regarded as an inverted pendulum, with the whole-body COM travelling from a trailing position to a leading position while the COP is controlled within a relatively small base of support. The reduction in RCIA in both the sagittal and frontal planes may indicate inadequate linear and angular velocities of the inverted pendulum for the COM to progress over the stationary foot (Pai and Patton, 1997; Wu et al., 2020; Lee P. A. et al., 2021; Lee et al., 2022), resulting in the loss of balance, thus increasing the risk of falling. While the LPPA allows minimal muscle damage, preserves medial blood supply, and improves patellar tracking as compared to the traditional approach (Cristea et al., 2016; Gunst et al., 2016), the damaged quadriceps tendon and the separation of the vastus lateralis from the remainder of the quadriceps around the lateral border of the patella (Liu et al., 2001) can still result in patellar tracking disorders and difficulties in the function of the extensor mechanism (Bindelglass and Vince, 1996), contributing to the observed alterations in the whole-body balance control, especially in the frontal plane.

In contrast to LPPA, patients with MVA showed better recovery in the COM-COP control with most IA and RCIA variables similar to those of the healthy controls throughout the gait cycle, except for greater sagittal IA during DLS and greater frontal IA during SLS but with less sagittal RCIA during SLS. During DLS, the MVA group walked with close-to-normal COM-COP control despite the slight increase in the sagittal IA, indicating a normal body weight transfer. Since inadequate body weight transfer increases the risk of falls in the elderly (Robinovitch et al., 2013), a normal weight transfer for MVA suggests that patients who had undergone TKA *via* MVA were at a no greater fall risk than healthy controls. During SLS, the MVA group also showed improvement in balance control with normal frontal RCIA.

The better recovery of balance control in the MVA group was further confirmed by direct comparisons between the LPPA and MVA groups. Compared to the LPPA, the MVA showed better recovery in the COM-COP balance control as indicated by significantly greater sagittal range of IA and frontal average RCIA throughout the gait cycle. It is known that balance is positively correlated with quadriceps strength (Yahia et al., 2011; Güner et al., 2015). The quadriceps tendon and the quadriceps muscle group, namely, vastus lateralis, vastus medialis and rectus femoris, play an important role in lower-limb stability and balance-perturbed walking (Bui et al., 2018; Kim and Chou, 2022). For the current patients who had undergone TKA *via* MVA, the vastus medialis oblique muscle belly was split in the direction of its fibers, which may improve the blood supply to the patella without damaging the quadriceps tendon. With less damage to the knee extensor mechanism than the LPPA, the MVA procedure provided better patellar tracking and a better function of the extensor mechanism (Dalury and Jiranek, 1999; Nutton et al., 2014), contributing to the observed better recovery of the whole-body balance control.

This current study was the first to investigate the post-operative performance of the whole-body balance control during level walking between older people who had undergone TKA *via* LPPA vs. MVA, in terms of COM-COP IA and RCIA. The results are limited to patients 3 months post-surgery. Follow-ups beyond 3 months will be needed to provide further evidence for the long-term performance differences. The proportion of females and males in the current study groups is representative of the actual occurrence where knee OA is more prevalent in women compared to men (Srikanth et al., 2005). Further study will be needed to identify the possible effects of sex or sex-related differences in tibiofemoral and Q angles on the whole-body balance control during level walking in older people with knee OA. Further studies using the current approach are also suggested for insight into how other surgical approaches might affect balance control during walking in the older population. For those with unilateral knee OA or valgus knee patients, the COM control relative to the COP is expected to be different from the current patients’, especially in the frontal plane. Further study will also be needed to identify the differences in unilateral TKA *via* LPPA vs. MVA on balance control during level walking. The current study assessed the participants’ balance control abilities while they walked at their preferred speeds, indicative of their physical and control capabilities. To fully understand the impact of gait speed on balance control, further study will be needed to investigate slower and faster walking speeds than the participants’ preferred speeds.

## Conclusion

The study compared the whole-body balance control during level walking in older people 3 months after TKA for medial knee OA *via* LPPA or MVA. The patients with LPPA showed compromised balance control during weight transfer and single-limb support in both sagittal and frontal planes. In contrast, patients with MVA showed better recovery in balance control, with most IA and RCIA variables back to normal throughout the gait cycle. The observed differences between LPPA and MVA in balance control were likely to be related to the differences between the surgical approaches such as the muscles involved. The current results suggest that MVA may be a better choice than LPPA for TKA when considering gait balance control in the frontal plane. Further study will be needed to identify the effects of MVA and LPPA on balance control in patients with unilateral knee OA.

## Data Availability

The original contributions presented in the study are included in the article/Supplementary Material, further inquiries can be directed to the corresponding authors.
